# Effect of implementing quality control management in the treatment of severely injured patients: a retrospective cohort study in a level I trauma center in China

**DOI:** 10.1186/s12873-022-00595-8

**Published:** 2022-03-05

**Authors:** Zhe Du, Tianbing Wang

**Affiliations:** Trauma Center, Peking University People’s Hospital, Beijing, 100044 China

**Keywords:** Severe trauma, Multidisciplinary team, Quality control management, Mortality, Emergency operation

## Abstract

**Background:**

This study aimed to review the impact of quality control management on the treatment of severely injured patients.

**Methods:**

A retrospective analysis was conducted on patients with severe injury (injury severity score [ISS] ≥ 16) between January 1, 2018 and February 1, 2020. The selected patients were stratified as follows. The patients who were admitted prior to the implementation of quality control management—from January 1 to December 31, 2018—were assigned to the PRE group; the POST group included patients who were admitted after the implementation—from February 1, 2019 to February 1, 2020. Quality control management was implemented from January 1, 2019 to January 31, 2019. Parameters were compared to account for differences in terms of demographics, surgical procedures, results of process quality, and 72-h mortality.

**Results:**

This study included 599 patients (PRE group: 212 males and 86 females; POST group: 228 males and 73 females; *P* = 0.20). The extent of document completion was 97.3 and 100% in the PRE and POST groups, respectively (*P* < 0.001). There was no delay in the arrival of the trauma surgeons or the multidisciplinary team after implementation. However, following implementation of quality control management, there was a significant reduction in the duration of basic diagnostics, time until receipt of laboratory data, time until first computed tomography scan, time until intubation, and time until an emergency operation (*P* < 0.05). The deaths were caused by severe head injury (PRE: 5.4%, POST: 4%), hemorrhagic shock (PRE: 2.4%, POST: 0.7%), multiple-organ failure (PRE: 1.0%, POST: 0.3%), or other causes (PRE: 0.7%, POST: 0.0%). The 72-h mortality decreased after the implementation of quality control management (PRE vs. POST groups: 9.4 vs. 5.0%, *P* = 0.04).

**Conclusions:**

The implementation of quality control management resulted in decreased time to critical interventions, improved patient care efficiency, and reduced early mortality. We recommend that this approach be replicated at other trauma centers in China.

**Supplementary Information:**

The online version contains supplementary material available at 10.1186/s12873-022-00595-8.

## Introduction

Injury, particularly road traffic injury, is a leading cause of death and disability among young people in China, according to a report published by the Chinese Ministry of Health in 2016 [1]. In high income countries, trauma centers help thousands of injured individuals every day and play a critical role in the response to disasters [2]. A trauma center implements treatment guidelines, establishes a multidisciplinary trauma team, holds expert conferences, and initiates quality improvement programs, which potentially contribute to the reduction in the rates of preventable morbidity and mortality [3]. Data from the United States demonstrated that up to 40% of deaths from injuries could be prevented with uniform access to well-organized systems of trauma care throughout the country [4]. In 2002, there were 1154 trauma centers in the United States, and the number of level I and II centers per million population ranges from 0.19 to 7.8 by state [2]. However, unlike in the developed countries of Europe as well as in the United States, the construction of trauma centers in China started much later, specifically in the year 2015 [5]. Trauma networks was first developed by the Peking University Trauma Medicine Center which could serve as a template for the rest of the country [1]. By the end of 2018, trauma treatment systems and trauma centers had been implemented in 431 hospitals in 28 provinces covering 55 cities and nearly 200 million people [6].

In addition to a multidisciplinary trauma team, quality control of trauma care is essential to verify the effectiveness of trauma centers and trauma systems [7]. In early 2006, the American College of Surgeons Committee on Trauma initiated the trauma quality improvement program (TQIP) as the next step in the improvement of the quality of care administered in trauma centers [8]. Additionally, a multidisciplinary quality management system (MQMS) was developed in Germany for improvements in the clinical treatment of severely injured patients. The quality assessment criteria were defined with respect to the timely appearance of the team, diagnostics, and timely and adequate therapy [3].

While these trauma centers have been developed with relatively complete multidisciplinary trauma teams [6], the quality control criteria for trauma management in China still requires some improvement and further development. Until now, there is no consensus of the quality control criteria for trauma centers in China. Furthermore, the effects of implementing quality control management on the patient outcomes in trauma centers are unknown. In our center, a quality management project was implemented in 2019 in order to deliver better medical services. In this project, we established ten assessment criteria for quality control management based on recommendations from the China Trauma Treatment Alliance in order to improve therapeutic effectiveness and outcome. Therefore, in this study, we aimed to (i) determine whether the implementation of a quality control management program can lead to significant improvements in process quality, (ii) determine whether the implementation of quality control management results in decreased 72-h mortality in the treatment of severely injured patients (injury severity score [ISS] ≥ 16), (iii) recommend standardized quality control management practices which can be promoted in more trauma centers in China. This study and its findings may help assess if there are any improvements in the quality of medical services rendered to patients, which in turn would yield better health outcomes, after evaluating cases before and after the implementation of the quality improvement program for trauma management.

### Patients and methods

#### Inclusion and Exclusion Criteria

**Fig. 1:**
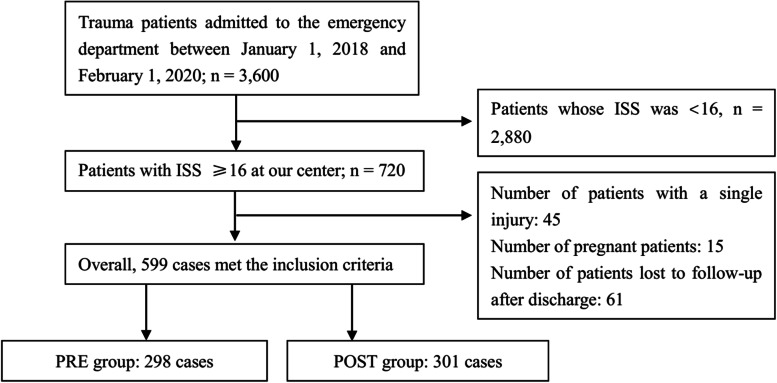
Flowchart showing the process of patient enrolment and exclusion

The patients’ records were retrieved retrospectively from our hospital’s patient registry. The selection criteria were as follows: a) age > 18 years, b) ISS score ≥ 16, and c) admission between January 1, 2018 and February 1, 2020. The exclusion criteria were as follows: a) children (under or equal to 18 years of age) and b) pregnant women. The patient screening process is shown in Fig. 1.

This was a retrospective study that evaluated the effect of quality control management on the treatment of patients with severe trauma (ISS ≥ 16). Quality control management was implemented from January 1, 2019 to January 31, 2019. Based on whether the patients were admitted before or after the implementation of quality control management, they were categorized into either the PRE group (admitted prior to the implementation, i.e., between January 1 and December 31, 2018) or the POST group (admitted after the implementation, i.e., between February 1, 2019 and February 1, 2020). This study was approved by the Institutional Review Board of the Peking University People’s Hospital.

### Trauma service

Early treatment in suspected severe trauma was administered in the resuscitation room of the emergency department. A qualified team of trauma surgeons, anesthesiologists, and laboratory and blood bank personnel were available throughout (Supplemental Table 1). The initial assessment and rescue of trauma patients were in strict accordance with the principles of the advanced trauma life support (ATLS). Activation of the trauma team was protocol-driven according to the traumatic event. Any of the following may initiate the activation of the trauma team: a) Systolic arterial pressure < 90 mm Hg; b) Respiratory rate < 9 or > 29 breaths/min; c) Heart rate > 120 beats/min; d) Glasgow Coma Scale (GCS) score < 14; and e) Trauma events including penetrating injuries, fractures, traumatic amputations, blast or crush injuries, major burns, motor vehicle crashes, falls, and helicopter emergency medical service transfers [9]. On each activation in either the PRE or POST groups, a minimum number of members of the trauma team must arrive; this includes 1 trauma surgeon (attending or associate chief of doctors), 1 critical medicine physician (attending or associate chief of doctors), and 1 trauma center resident (Supplemental Table 1). The senior-most doctor serves as the team leader. Depending on the patient’s situation, the leader selectively calls trauma team members from the appropriate departments. Although the study period coincided with the onset of the COVID-19 pandemic, this did not affect trauma activation and team composition in the POST group.

**Table 1:** Assessment criteria for quality control management

1. Completeness of documentation: less than 10% of missing data in the emergency department protocol
2. Arrival of the trauma surgeon: arrival on time with (or before) the patient
3. Arrival of the multidisciplinary team: arrival on time with (or before) the patient
4. Time for basic diagnostics in severe trauma: within 20 min of hospital admission
5. Time until data on the level of hemoglobin: within 30 min of hospital admission
6. Time until whole-body CT in severe trauma: within 30 min of hospital admission
7. Time until intubation when GCS < 9, systolic blood pressure < 90 mmHg, respiratory insufficiency: within 10 min of hospital admission
8. Time until chest tube insertion in cases with clinical/radiological manifestations of hemothorax/pneumothorax or other cases, as determined by the team leader: within 30 min of hospital admission
9. Time until transfusion in case of initial hemorrhagic shock (hemoglobin < 8 g/dL, systolic blood pressure < 90 mmHg): within 30 min of hospital admission
10. Time until emergency operation (determined by the trauma team leader): within 120 min of hospital admission

*CT* Computed tomography, *GCS* Glasgow coma scale

### Intervention

The implementation of quality control management included the provision of the assessment criteria to the trauma team and ensuring the test team members’ familiarity with these criteria. The full score was 100, and a score of 90 was regarded as qualified for mastering these criteria. The assessment criteria of quality control management are summarized in Table 1.

### Establishing a quality improvement (QI) system

The QI system included all members of the trauma team and the heads of all departments involved in trauma care (Supplemental Table 1). The head of the trauma center directed the QI program. A dedicated trauma registry database was used, and one database administrator collected and processed the data. Quality control meetings were conducted on a weekly basis. Structural or organizational changes were analyzed by evaluating the prospectively collected data, and the trauma team members and staff were notified about any changes in the protocol during the meetings.

### Data collection

Data on the demographics, surgical procedures, results of process quality, and 72-h mortality in our center were collected and analyzed.

### Statistical analyses

Data are expressed as means (standard deviation) or as numbers (percentages). Statistical tests were performed using SPSS 14.0 for Windows (SPSS, Inc., Chicago, IL). The Chi-square test was used for analyzing categorical data. A *P*-value of < 0.05 was considered statistically significant.

## Results

### Patient characteristics

After screening for eligibility, 599 patients were included in this study (PRE group: 212 males, 86 females; POST group: 228 males, 73 females; *P* = 0.20). The mean (SD) ages were 52.3 (16.4) and 50.4 (15.4) years in the PRE and POST groups, respectively, *P* = 0.16. Data on other parameters including the patient source, mechanism of injury, ISS score, GCS score, numbers of whole body-CT, intubation, chest tube insertion, transfusion, surgery before intensive care unit (ICU) admission, duration of ICU admission, length of stay, and ventilator usage time are listed in Table 2. There were no significant differences in the aforementioned parameters between the PRE and POST groups (*P* > 0.05) (Table 2).

**Table 2:** Baseline characteristics of severely injured patients (ISS ≥ 16)

Items	PRE group	POST group	*P*-value
Age (years)	52.3 (16.4)	50.4 (15.4)	0.16
Sex	298	301	0.20
Male	212 (71.1%)	228 (79.1%)	
Female	86 (28.9%)	73 (24.3%)	
Direct admission	173 (58.1%)	173 (57.5%)	0.89
Transferred from another hospital	125 (42.0%)	128 (42.5%)	0.89
Mechanism of injury			-
Motor vehicle crash	150 (50.3%)	150 (49.8%)	
Fall	97 (32.6%)	86 (28.6%)	
Penetrating trauma	14 (4.7%)	15 (5.0%)	
Others	37 (12.4%)	50 (16.6%)	
ISS	24.3 (8.5)	24.9 (8.5)	0.46
16–25	189 (63.4%)	190 (63.1%)	
26–35	75 (25.2%)	77 (25.9%)	
36–50	31 (10.4%)	32 (10.6%)	
> 51	3 (1.0%)	2 (0.7%)	
GCS	12.6 (3.9)	12.6 (3.6)	0.92
3–8	71 (23.8%)	64 (21.3%)	
9–12	55 (18.5%)	39 (13.0%)	
13–15	172 (57.7%)	198 (65.8%)	
Whole-body CT	279 (93.6%)	281 (93.4%)	0.89
Intubation	60 (20.1%)	58 (19.3%)	0.79
Chest tube insertion	54 (18.1%)	58 (19.3%)	0.72
Transfusion	157 (52.7%)	152 (50.5%)	0.59
Surgery before ICU admission	97 (32.6%)	108 (35.9%)	0.39
ICU stay (hours)	239.5 (262.1)	192.9 (244.6)	0.09
Length of stay (days)	25.6 (30.1)	22.3 (16.3)	0.12
Ventilator usage time (hours)	152.1 (235.9)	120.2 (144.4)	0.23

Data are expressed as means (standard deviation) or as numbers (percentages)

*CT* Computed tomography, *ISS* Injury severity score, *GCS* Glasgow coma scale, *ICU* Intensive care unit

### Process quality

The completeness of documentation was 97.3 and 100% in the PRE and POST groups, respectively (*P* < 0.001). There was no delay in the arrival of the trauma surgeons or the multidisciplinary trauma team after the implementation of quality control management. The duration of basic diagnostics, time until receipt of laboratory information, time until the first whole-body computed tomography (CT) scan, time until intubation, and time until an emergency operation decreased significantly after implementation (*P* < 0.05) (Table 3).

**Table 3:** Results of process quality assessment before and after intervention

Items	PRE group	POST group	*P*-value
Completeness of documentation	290/298 (97.3%)	301/301 (100%)	< 0.001
Delayed arrival of trauma surgeon	37/298 (12.4%)	0	
Time of delay (min)	7.0 (1.3)	-	
Delayed arrival of MDT	37/298 (12.4%)	0	
Time of delay (min)	6.3 (1.2)	-	
Duration of basic diagnostics (min)	24.0 (17.6)	13.5 (6.0)	< 0.001
Duration until data on the hemoglobin level were available (min)	59.6 (43.6)	50.8 (41.0)	0.04
Duration until first CT scan (min)	44.2 (23.4)	27.9 (17.8)	< 0.001
Time until intubation (min)	35.9 (33.0)	21.1 (9.4)	0.02
Time until chest tube insertion (min)	102.9 (100.3)	55.9 (17.3)	0.11
Time until transfusion (min)	96.7 (77.6)	48.9 (23.4)	< 0.001
Time until emergency operation (min)	123.9 (14.3)	120.6 (12.3)	0.86

Data are expressed as means (standard deviation) or as numbers (percentages)

*MDT* Multidisciplinary team, *CT* Computed tomography, Not applicable

### Patient outcomes

The causes of death were severe head injuries (PRE and POST groups: 5.4 and 4%, respectively); hemorrhagic shock (PRE and POST groups: 2.4 and 0.7%, respectively); multiple-organ failure (PRE and POST groups: 1.0 and 0.3%, respectively); and other causes (PRE and POST groups: 0.7 and 0%, respectively). The 72-h mortality decreased after the implementation (PRE vs. POST groups: 9.4 vs. 5.0%, *P* = 0.04) (Table 4).

**Table 4:** Comparison of 72-h mortality in severe trauma patients (ISS ≥ 16) before and after intervention

Items	PRE group	POST group	*P*-value
Cause of death			-
Severe head injury	16/298 (5.4%)	12/301 (4.0%)	-
Hemorrhagic shock	7/298 (2.4%)	2/301 (0.7%)	-
Multiple-organ failure	3/298 (1.0%)	1/301 (0.3%)	-
Other causes	2/298 (0.7%)	0 (0.0%)	-
Mortality within 72 h	28/298 (9.4%)	14/301 (5.0%)	0.04

Data are expressed as numbers (percentages)

## Discussion

This study was a retrospective investigation into the effect of implementing quality control management in the treatment of severely injured patients. Our results showed that the implementation of quality control management can decrease the time to critical interventions, improve patient care efficiency, and reduce early mortality.

The aim of a trauma system is to facilitate the timely treatment of severely injured patients with available resources for their optimal management and rehabilitation [10]. Trauma treatment systems in China were developed in 2015, and did not commence as early as that in Europe and America. The China Trauma Treatment Alliance developed trauma treatment systems and trauma centers throughout the country. By the end of 2018, this initiative had been implemented in 431 hospitals across 28 provinces covering 55 cities and nearly 200 million people. Moreover, the national health commission implemented a series of policies throughout China to improve the ability of the medical staff to treat severe trauma [6]. A previous multi-center study in China reported that the mortality rate of patients with severe trauma (ISS ≥ 16) decreased from 33.82 to 20.49% after the development of trauma centers [11].

Although quality control is pervasive in most modern businesses, it is in its infancy in medicine [12]. Based on the development of trauma centers, we wanted to assess whether the implementation of quality control could further improve treatment efficiency and reduce mortality. In our study, the process quality was improved after the implementation of the quality control management project. The time to critical interventions (such as time to the first CT scan, time until intubation, and time until transfusion) was decreased after the intervention. With an ameliorated treatment process, there was also a trend towards better outcomes. A previous study identified a correlation between the quality management system and improved treatment [3]. Several authors have also noted the negative impact of delayed diagnoses on outcomes after severe trauma [13]. However, some process quality indicators did not meet the standard requirements in the project. For example, the average time until intubation was 21.1 min (assessment criterion: < 10 min) and the average time until transfusion was 48.9 min (assessment criterion: < 30 min). Reasons could include inadequate cooperation between team members, an imperfect process system, or inadequate training of doctors. The results indicated that interaction and regular communication among the involved members need further improvement in the future. We also noticed that there were no significant differences in terms of the duration of ICU stay, length of stay, and ventilator usage time before and after intervention (*p* > 0.05) in this study. Nevertheless, the 72-h mortality rate had decreased significantly to 5.0% (*p* = 0.04) after the intervention.

There were some limitations in this study. First, the sample size was limited, and our findings need to be validated in larger trials. Second, this study was not a randomized controlled trial, and thus, the improvements in time to critical operation and decreased mortality cannot be directly attributed to quality control management [14]. The relationships between trauma team efficiency and decreased early mortality and influencing factors need to be investigated further. Third, this was a single-center study. A multi-center study should be performed in the future to validate the findings of this study.

## Conclusions

The implementation of quality control management reduced the time to critical interventions, improved patient care efficiency, and reduced early mortality. We recommend that this approach be replicated at other trauma centers in China.

## Supplementary Information

**Additional file 1.**

## Data Availability

The datasets generated and analyzed during the current study are not publicly available due to these data were used under license for the current study but are available from the corresponding author on reasonable request.

## Abbreviations

TQIP: Trauma quality improvement program
MQMS: Multidisciplinary quality management system
ATLS: Advanced trauma life support
GCS: Glasgow Coma Scale
