# Supplementary material for: Effect of implementing quality control management in the treatment of severely injured patients: a retrospective cohort study in a level I trauma center in China
Source: BMC Emerg Med. 2022 Mar 5;22:34. doi: 10.1186/s12873-022-00595-8 (PMC8897735; doi:10.1186/s12873-022-00595-8)
Supplement: Supplementary file 1 — Additional file 1. [file 12873_2022_595_MOESM1_ESM.docx]

Supplemental Table 1Trauma team: Departments and personnel

| Department | Personnel |
| --- | --- |
| Trauma Center | 1 trauma surgeon, 1 critical medicine physician, 2 ED nurses, 1 resident ^a^ |
| General surgery | 1 general surgeon ^b^ |
| Neurosurgery | 1 neurosurgeon ^b^ |
| Thoracic surgery | 1 thoracic surgeon ^b^ |
| Urology Surgery | 1 urology surgeon ^b^ |
| Vascular surgery | 1 vascular surgeon ^b^ |
| Anesthesiology | 1 anesthesiologist, 1 or 2 nurses |
| Radiology | 1 radiologist, 1 radiology technician |
| Laboratory | 24-hour laboratory technicians |
| Blood bank | 24-hour laboratory technicians |
| Consultant specialties | Maxillofacial surgery, otorhinolaryngology, ophthalmology ^c^ |

ED = emergency department

^a^ The trauma surgery team is directly available and serve patients 24/7.

^b^ The surgeons are called in cases of suspected severe abdominal trauma, traumatic brain injury, or other such severe conditions. The surgeon may have to be called from home (i.e., on stand-by duty).

^c^ The attending surgeon may have to be called from home (i.e., on stand-by duty).
